# Synthesis and Characterization of New Palladium(II) Thiosemicarbazone Complexes and Their Cytotoxic Activity against Various Human Tumor Cell Lines

**DOI:** 10.1155/2013/524701

**Published:** 2013-12-11

**Authors:** Wilfredo Hernández, Juan Paz, Fernando Carrasco, Abraham Vaisberg, Evgenia Spodine, Jorge Manzur, Lothar Hennig, Joachim Sieler, Steffen Blaurock, Lothar Beyer

**Affiliations:** ^1^Facultad de Ingeniería Industrial, Universidad de Lima, Avenida Javier Prado Este Cuadra 46, Urbanización Monterrico, Lima 33, Peru; ^2^Facultad de Ciencias Naturales y Matemática, Universidad Nacional Federico Villarreal, Jr. Río Chepén s/n, El Agustino; Lima, Peru; ^3^Laboratorio de Investigación y Desarrollo, Facultad de Ciencias y Filosofía, Universidad Peruana Cayetano Heredia, Avenida Honorio Delgado 430, Urbanización Ingeniería-San Martin de Porras, Lima 31, Peru; ^4^Facultad de Ciencias Químicas y Farmacéuticas, Universidad de Chile, CEDENNA, Olivos 1007, Casilla 233, Independencia, 8330492 Santiago, Chile; ^5^Facultad de Ciencias Físicas y Matemáticas, Universidad de Chile, CEDENNA, 8370448 Santiago, Chile; ^6^Fakultät für Chemie und Mineralogie, Universität Leipzig, Johannisallee 29, 04103 Leipzig, Germany

## Abstract

The palladium(II) bis-chelate complexes of the type [Pd(TSC^1-5^)_2_] (**6–10**), with their corresponding ligands 4-phenyl-1-(acetone)-thiosemicarbazone, HTSC^1^ (**1**), 4-phenyl-1-(2′-chloro-benzaldehyde)-thiosemicarbazone, HTSC^2^ (**2**), 4-phenyl-1-(3′-hydroxy-benzaldehyde)-thiosemicarbazone, HTSC^3^ (**3**), 4-phenyl-1-(2′-naphthaldehyde)-thiosemicarbazone, HTSC^4^ (**4**), and 4-phenyl-1-(1′-nitro-2′-naphthaldehyde)-thiosemicarbazone, HTSC^5^ (**5**), were synthesized and characterized by elemental analysis and spectroscopic techniques (IR and ^1^H- and ^13^C-NMR). The molecular structure of HTSC^3^, HTSC^4^, and [Pd(TSC^1^)_2_] (**6**) have been determined by single crystal X-ray crystallography. Complex **6** shows a square planar geometry with two deprotonated ligands coordinated to Pd^II^ through the azomethine nitrogen and thione sulfur atoms in a *cis* arrangement. The *in vitro* cytotoxic activity measurements indicate that the palladium(II) complexes (IC_50_ = 0.01–9.87 *μ*M) exhibited higher antiproliferative activity than their free ligands (IC_50_ = 23.48–70.86 and >250 *μ*M) against different types of human tumor cell lines. Among all the studied palladium(II) complexes, the [Pd(TSC^3^)_2_] (**8**) complex exhibited high antitumor activity on the DU145 prostate carcinoma and K562 chronic myelogenous leukemia cells, with low values of the inhibitory concentration (0.01 and 0.02 *μ*M, resp.).

## 1. Introduction

In recent years, sulfur containing ligands such as dithiocarbamates and thiosemicarbazones and their transition metal complexes have received more attention in the area of medicinal chemistry, due to their pharmacological properties, such as antiviral [1–3], antibacterial [4–7], antifungal [8–10], antiparasitic [11, 12], and antitumor [13–19] activities.

The synthesis of thiosemicarbazones (R–CH=N–NH–CS–NHR^1^) has been developed due to the facility to replace the R and R^1^ substituent groups by alkyl, aryl, or heterocyclic derivative and thus leading to a broad spectrum of new bidentate (N,S or N,N) and tridentate (N,N,N or N,N,S) and also tetra- and pentadentate ligands, capable of coordinating to metal centres [6, 20–22].

It has been shown that the *α*-(N)-heterocyclic carbaldehyde thiosemicarbazones act as chelating agents of the transition metals and some of them exhibit antitumor activity by inhibiting the biosynthesis of DNA, possibly by blocking the enzyme ribonucleotide diphosphate reductase [23–25]. On the other hand, the ligand 6-methylpyridine-2-carbaldehyde-N(4)-ethylthiosemicarbazone (HmpETSC) and its complexes [Zn(HmpETSC)Cl_2_] and [Pd(mpETSC)Cl] exhibit antineoplastic activity against colon cancer human cell lines (HCT 116) with IC_50_ values of 14.59, 16.96, and 20.65 *μ*M, respectively [26].

In previous articles, we have reported the cytotoxic activity of the ligands derived from benzaldehyde and furaldehyde thiosemicarbazone and their palladium(II) bis-chelate complexes. *In vitro* antitumor studies against different human tumor cell lines revealed that these metal complexes (IC_50_ = 0.21–12.46 *μ*M) were more cytotoxic than their corresponding ligands (IC_50_ > 60 *μ*M). On the other hand, the platinum(II) tetranuclear, [Pt_4_L_4_] (HL = 4-phenyl-1-benzaldehyde thiosemicarbazone), exhibits higher antiproliferative activity with IC_50_ values in the range of 0.07–0.12 *μ*M [27].

The present work describes the synthesis, characterization, and antitumor activity of palladium(II) bis-chelate complexes of the type [Pd(TSC^1–5^)_2_] (**6**–**10**) with the ligands 4-phenyl-1-(acetone)-thiosemicarbazone, HTSC^1^ (**1**), 4-phenyl-1-(2′-chloro-benzaldehyde)-thiosemicarbazone, HTSC^2^ (**2**), 4-phenyl-1-(3′-hydroxy-benzaldehyde)-thiosemicarbazone, HTSC^3^ (**3**), 4-phenyl-1-(2′-naphthaldehyde)-thiosemicarbazone, HTSC^4^ (**4**), and 4-phenyl-1-(1′-nitro-2′-naphthaldehyde)-thiosemicarbazone, HTSC^5^ (**5**).

## 2. Experimental

### 2.1. Materials and Measurements

Chemicals were reagent grade and were used without further purification. Palladium(II) bis(acetylacetonate), potassium tetrachloropalladate, acetone, 4-phenyl-thiosemicarbazide, o-chloro-benzaldehyde, m-hydroxy-benzaldehyde, naphthaldehyde, and 1-nitro-2-naphthaldehyde were purchased from Aldrich. Elemental analyses were determined on a Fisons-Carlo Erba Elemental Microanalyzer. Infrared spectra were recorded as KBr pellets (4000–400 cm^−1^) on a Bruker FT-IR IFS 55 Equinox spectrophotometer. The FAB(+) mass spectra were recorded on a ZAB-HSQ (V.G. Analytical Ltd. Floats Roads, Wythenshawe, Manchester, UK) spectrometer, using 3-nitrobenzyl alcohol as the matrix. NMR spectra were recorded on a Bruker Avance DRX 300 spectrometer in DMSO-d_6_, operating at 300 and 75.5 MHz (^1^H, ^13^C). The chemical shifts were measured in ppm relative to tetramethylsilane (SiMe_4_).

### 2.2. Synthesis of the Ligands

#### 2.2.1. General Method

To a hot solution of 4-phenyl thiosemicarbazide (3.34 g, 20 mmol) in methanol (100 mL) was added a solution of acetone (1.47 mL, 20 mmol) in 40 mL of methanol with a few drops of glacial acetic acid. The reaction mixture was refluxed for 2-3 h and stirred for 24 h at room temperature. The solid product was filtered, washed several times with ethanol, and dried *in vacuo*. A similar procedure was applied using o-chloro-benzaldehyde (2.25 mL, 20 mmol) in 60 mL of methanol, m-hydroxy-benzaldehyde (2.44 g, 20 mmol) in 60 mL of methanol, naphthaldehyde (2.72 mL, 20 mmol) in 40 mL of methanol, or 1-nitro-2-naphthaldehyde (4.02 g, 20 mmol) in 70 mL of methanol. Single crystals suitable for X-ray crystallography for both HTSC^3^ and HTSC^4^ were obtained by slow evaporation of the solvent at room temperature.

#### 2.2.2. 4-Phenyl-1-acetone Thiosemicarbazone, HTSC^1^ (**1**)

Colorless solid. Yield 78%. Anal. for C_10_H_13_N_3_S (207.30 g/mol): calcd. C 57.94, H 6.32, N 20.27, S 15.47; found C 58.07, H 6.48, N 20.09, S 15.40. FAB(+)-MS: *m*/*z* 207.3 (M^+^, 100%). IR (KBr): *ν* = 3251 (NHPh), 3182 (NHCS), 1600 (C=N), 820, 1078 (C=S) cm^−1^. ^1^H NMR (DMSO-d_6_): **δ** = 2.0 (s, CH_3_); 7.16 (t, 1H_para_, NHPh, *J *= 7.5 Hz), 7.33 (t, 2H_meta_, NHPh, *J *= 8.1 Hz), 7.61 (d, 2H_ortho_, NHPh, *J *= 7.5 Hz), 9.83 (s, 1H, NHPh); 10.35 (s, 1H, =N–NH). ^13^C NMR (DMSO-d_6_): **δ** = 18.39, 25.54 (CH_3_), 125.36, 128.48, 130.48, 139.52 (NHPh); 153.21 (HC=N); 176.78 (C=S).

#### 2.2.3. 4-Phenyl-1-(2′-chlorobenzaldehyhe) Thiosemicarbazone, HTSC^2^ (**2**)

Yellow solid. Yield 72%. Anal. for C_14_H_12_N_3_ClS (289.79 g/mol): calcd. C 58.03, H 4.17, N 14.50, Cl 12.23, S 11.07; found C, 57.92, H 4.04, N 14.73, Cl 12.15, S 11.21. FAB(+)-MS: *m*/*z* 290.70 (MH^+^, 100%). IR (KBr): *ν* = 3305 (NHPh), 3166 (NHCS), 1600 (C=N), 835, 1065 (C=S) cm^−1^. ^1^H NMR (DMSO-d_6_): **δ** = 8.46 (d, H^3^′, *J *= 7.5 Hz), 7.50 (m, H^4^′), 7.22 (t, H^5^′, *J *= 7.2 Hz), 7.33 (d, H^6^′, *J *= 8.0 Hz); 7.58 (d, 2H_ortho_, NHPh, *J *= 8.7 Hz), 7.38 (t, 2H_meta_, NHPh, *J *= 8.4 Hz), 7.16 (t, 1H_para_, NHPh, *J* = 7.5 Hz); 8.59 (s, 1H, HC=N); 10.22, 9.83 (s, 1H, NHPh); 12.03, 10.35 (s, 1H, =N–NH). ^13^C NMR (DMSO-d_6_): **δ** = 124.78, 126.47, 128.29, 129.17, 130.98, 133.78 (Ph–CH=N–); 125.37, 128.48, 130.29, 139.38 (NHPh); 153.22 (HC=N); 176.92 (C=S).

#### 2.2.4. 4-Phenyl-1-(3′-hydroxybenzaldehyde) Thiosemicarbazone, HTSC^3^ (**3**)

Colorless crystals. Yield 87%. Anal. for C_14_H_13_N_3_OS (271.34 g/mol): calcd. C 61.97, H 4.83, N 15.49, S 11.82; found C 60.65, H 4.95, N 15.16, S 11.64. FAB(+)-MS: *m*/*z* 272.25 (MH^+^, 100%). IR (KBr): *ν* = 3290 (NHPh), 3140 (NHCS), 1598 (C=N), 825, 1020 (C=S) cm^−1^. ^1^H NMR (DMSO-d_6_): **δ** = 7.36 (m, H^2^′), 7.21 (m, H^4^′), 7.09 (m, H^5^′), 7.41 (d, H^6^′, *J *= 7.8 Hz); 7.57 (d, 2H_ortho_, NHPh, *J *= 7.5 Hz), 7.33 (t, 2H_meta_, NHPh, *J *= 8.1 Hz), 7.15 (t, 1H_para_, NHPh, *J* = 7.2 Hz); 8.07 (s, 1H, HC=N); 9.56 (s, 1H, OH); 10.34, 9.91 (s, 1H, NHPh), 11.77, 10.07 (s, 1H, =N–NH). ^13^C NMR (DMSO-d_6_): **δ** = 114.68, 118.93, 125.80, 130.05, 135.32, 158.02 (Ph–CH=N–); 121.84, 125.31, 128.06, 139.1 (NHPh); 152.79 (HC=N); 193.17 (C=S).

#### 2.2.5. 4-Phenyl-1-naphthaldehyde Thiosemicarbazone, HTSC^4^ (**4**)

Rectangular-shaped yellow crystals. Yield 75%. Anal. for C_18_H_15_N_3_S (305.39 g/mol): calcd. C 70.79, H 4.95, N 13.76, S 10.50; found: C 70.93, H 4.80, N 13.85, S 10.38. FAB(+)-MS: *m*/*z* 305.40 (M^+^, 100%). IR (KBr): *ν* = 3327 (NHPh), 3165 (NHCS), 1600 (C=N), 815, 1088 (C=S) cm^−1^. ^1^H NMR (DMSO-d_6_): **δ** = 7.33 (d, H^2^′, 7.8 Hz), 7.67 (t, H^3^′, 7.2 Hz), 8.34 (d, H^4^′, 8.4 Hz), 8.47 (d, H^5^′, H^8^′, *J *= 6.0 Hz), 7.67 (t, H^6^′, *J *= 7.2 Hz), 8.02 (t, H^7^′, *J *= 7.2 Hz); 7.39 (t, 2H_meta_, NHPh, *J *= 7.8 Hz), 7.16 (t, 1H_para_, NHPh, *J* = 7.2 Hz), 7.61 (d, 2H_ortho_, NHPh, *J *= 7.2 Hz); 9.08 (s, 1H, HC=N); 9.84, 10.35 (s, 1H, NHPh); 10.20, 11.89 (s, 1H, =N–NH). ^13^C NMR (DMSO-d_6_): **δ** = 122.94, 125.83, 126.05, 126.62, 127.69, 129.37, 130.87, 131.15, 133.83, 141.45 (Naphthoyl); 125.38, 126.25, 128.57, 139.54 (NHPh), 153.25 (HC=N); 176.38 (C=S).

#### 2.2.6. 4-Phenyl-1-(1′-nitro-2′-naphthaldehyde) Thiosemicarbazone, HTSC^5^ (**5**)

Yellow solid. Yield 85%. Anal. for C_18_H_14_N_4_O_2_S (350.39 g/mol): calcd. C 61.69, H 4.03, N 16.00, S 9.15; found: C 61.54, H 4.10, N 15.82, S 8.94. FAB(+)-MS: *m*/*z* 350.40 (M^+^, 100%). IR (KBr): *ν* = 3250 (NHPh), 3174 (NHCS), 1713, 1626 (C=N), 820, 1047 (C=S) cm^−1^. ^1^H NMR (DMSO-d_6_): **δ** = 8.40 (d, H^3^′, *J *= 8.5 Hz), 8.09 (d, H^4^′, *J *= 8.5 Hz), 8.22 (d, H^5^′, H^8^′, *J *= 6.0 Hz), 7.86 (t, H^6^′, H^7^′, *J *= 6.0 Hz); 7.61 (d, 2H_ortho_, NHPh, *J *= 8.5 Hz), 7.33 (t, 2H_meta_, NHPh, *J *= 6.5 Hz), 7.15 (t, 1H_para_, NHPh, *J* = 7.5 Hz); 10.15 (s, 1H, NHPh), 9.83, 10.34 (s, 1H, =N–NH). ^13^C NMR (DMSO-d_6_): **δ** = 118.28, 123.71, 126.01, 129.17, 130.84, 132.22, 136.47, 137.96, 153.24, 163.47 (Naphthoyl); 122.65, 125.36, 128.49, 139.5 (NHPh); 176.76 (HC=N); 189.98 (C=S).

### 2.3. Synthesis of the Palladium(II) Complexes

#### 2.3.1. General Method

A solution of K_2_[PdCl_4_] (0.163 g, 0.5 mmol) in ethanol (60 mL) or a solution of [Pd(acac)_2_] (0.153 g, 0.5 mmol) in dichloromethane/ethanol (2 : 1, 45 mL) was added dropwise to a stirred hot solution of the corresponding thiosemicarbazone (1.0 mmol) in 70 mL of methanol. Then, sodium acetate (0.082 g, 1 mmol) in 5 mL of water was added. The solution was refluxed for 2-3 h and stirred for 24 h at room temperature. The precipitate was collected by filtration, washed three times with ethanol (30 mL), and dried under vacuum. For the complex [Pd(TSC^1^)_2_] (**6**), single crystals suitable for X-ray diffraction studies were grown by slow evaporation from an acetone solution.

#### 2.3.2. Bis[4-phenyl-1-(acetone) Thiosemicarbazonato]palladium(II), [Pd(TSC^1^)_2_] (**6**)

Square-shaped orange crystals. Yield 65%. Anal. for C_20_H_24_N_6_S_2_Pd (518.99 g/mol): calcd. C 46.28, H 4.66, N 16.19, S 12.36; found C 46.05, H 4.74, N 16.21, S 12.23. FAB(+)-MS: *m*/*z* 518.50 (M^+^, 75%). IR (KBr): *ν* = 3375 (NHPh), 1590 (C=N), 800, 964 (C=S) cm^−1^. ^1^H NMR (DMSO-d_6_): **δ** = 2.18, 2.35 (s, 12H, 4CH_3_); 7.62 (d, 4H_ortho_, *J* = 7.9 Hz, NHPh), 7.26 (t, 4H_meta_, *J* = 7.7 Hz, NHPh), 6.93 (t, 2H_para_, *J *= 7.5 Hz, NHPh); 9.34 (s, 2H, NHPh). ^13^C NMR (DMSO-d_6_): **δ** = 19.65, 22.08 (CH_3_), 119.06, 123.15, 129.01, 141.80 (NHPh); 154.50 (HC=N); 176.20 (C=S).

#### 2.3.3. Bis[4-phenyl-1-(2′-chlorobenzaldehyde) Thiosemicarbazonato]palladium(II), [Pd(TSC^2^)_2_] (**7**)

Red solid. Yield 60%. Anal. for C_28_H_22_N_6_Cl_2_S_2_Pd (683.97 g/mol): calcd. C 49.17, H 3.24, N 12.29, Cl 10.37, S 9.38; found C, 49.26, H 3.12, N 12.36, Cl 10.45, S 9.25. FAB(+)-MS: *m*/*z* 648.5 (M^+^-Cl, 65%). IR (KBr): *ν* = 3375 (NHPh), 1580 (C=N), 795, 927 (C=S) cm^−1^. ^1^H NMR (DMSO-d_6_): **δ** = 8.29 (d, H^3′^, *J *= 8.1 Hz), 7.52 (m, H^4′^), 7.28 (t, H^5′^,* J *= 7.8 Hz), 7.42 (d, H^6′^, *J *= 8.1 Hz); 7.59 (d, 4H_ortho_, NHPh, *J *= 8.1 Hz), 7.19 (t, 4H_meta_, NHPh, *J *= 8.1 Hz), 6.98 (t, 2H_para_, NHPh, *J* = 7.2 Hz); 8.0 (s, 2H, HC=N); 9.34, 9.92 (s, 1H, NHPh). ^13^C NMR (DMSO-d_6_): **δ** = 119.05, 120.83, 125.01, 126.72, 127.38, 138.77 (Ph–CH=N–); 121.94, 127.90, 129.0, 141.81 (NHPh); 166.48 (HC=N); 169.19 (C=S).

#### 2.3.4. Bis[4-phenyl-1-(3′-hydroxybenzaldehyde) Thiosemicarbazonato]palladium(II), [Pd(TSC^3^)_2_] (**8**)

Orange solid. Yield 68%. Anal. for C_28_H_24_N_6_O_2_S_2_Pd (647.08 g/mol): calcd. C 51.97, H 3.74, N 12.99, S 9.91; found C 52.05, H 3.66, N 13.04, S 9.83. FAB(+)-MS: *m*/*z* 646.95 (M^+^, 55%). IR (KBr): *ν* = 3300 (NHPh), 1585 (C=N), 800, 930 (C=S) cm^−1^. ^1^H NMR (DMSO-d_6_): **δ** = 7.02 (d, 2H^4′^, 2H^6′^,* J *= 8.4 Hz) 7.48 (t, 2H^5′^, *J *= 7.5 Hz); 7.60 (d, 4H_ortho_, NHPh, *J *= 7.8 Hz), 7.28 (t, 4H_meta_, NHPh, *J *= 8.4 Hz), 6.92 (t, 2H_para_, NHPh, *J* = 7.5 Hz); 7.81 (s, 2H, HC=N), 8.45 (d, OH,* J *= 7.5 Hz); 9.46 (s, 2H, NHPh,* J *= 7.5 Hz). ^13^C NMR (DMSO-d_6_): **δ** = 110.71, 121.75, 130.69, 133.47, 155.19 (Ph–CH=N–); 119.04, 120.0, 128.62, 141.23 (NHPh); 158.37 (HC=N); 167.06 (C=S).

#### 2.3.5. Bis[4-phenyl-1-naphthaldehyde Thiosemicarbazonato]palladium(II), [Pd(TSC^4^)_2_] (**9**)

Orange solid. Yield 61%. Anal. for C_36_H_28_N_6_S_2_Pd (715.20 g/mol): calcd. C 60.46, H 3.95, N 11.75, S 8.97; found: C 60.32, H 4.05, N 11.81, S 8.84. FAB(+)-MS: *m*/*z* 716.30 (MH^+^, 58%). IR (KBr): *ν* = 3373 (NHPh), 1588 (C=N), 780, 1032 (C=S) cm^−11^H NMR (DMSO-d_6_): **δ** = 8.44 (d, 2H^2′^, *J *= 9.0 Hz), 7.85 (m, 2H^3′^), 8.24 (d, 2H^4′^, 2H^5′^, *J *= 9.0 Hz), 7.85 (m, 2H^6′^, 2H^7′^), 8.14 (d, 2H^8′^, *J *= 6.0 Hz); 7.60 (d, 4H_ortho_, NHPh, *J *= 6.0 Hz), 7.33 (t, 4H_meta_, NHPh, *J *= 8.0 Hz), 7.15 (t, 2H_para_, NHPh, *J* = 8.5 Hz); 9.83, 10.15, 10.33 (s, 2H, NHPh). ^13^C NMR (DMSO-d_6_): **δ** = 118.05, 121.04, 123.64, 124.34, 126.12, 129.09, 132.50, 134.30, 136.47, 139.52 (Naphthoyl); 125.14, 126.77, 128.71, 141.04 (NHPh), 165.62 (HC=N); 175.72 (C=S).

#### 2.3.6. Bis[4-phenyl-1-(1′-nitro-2′-naphthaldehyde) Thiosemicarbazonato]palladium(II), [Pd(TSC^4^)_2_] (**10**)

Orange solid. Yield 50%. Anal. for C_36_H_26_N_8_O_4_S_2_Pd (805.19 g/mol): calcd. C 53.70, H 3.25, N 13.92, S 7.96; found: C 53.64, H 3.15, N 13.86, S 7.78. FAB(+)-MS: *m*/*z* 787.10 (M^+^-H_2_O, 100%). IR (KBr): *ν* = 3374 (NHPh), 1578 (C=N), 790, 1017 (C=S) cm^−1^. ^1^H NMR (DMSO, d_6_): **δ** = 7.80 (t, 2H^3′^, *J *= 9.0 Hz), 8.38 (d, 2H^4′^, 2H^5′^, *J *= 9.0 Hz), 7.80 (t, 2H^6′^, 2H^7′^, *J *=6.0 Hz), 7.31 (m, 2H^8′^); 7.62 (d, 4H_ortho_, NHPh, *J *= 9.0 Hz), 7.26 (t, 4H_meta_, NHPh, *J *= 9.0 Hz), 6.92 (t, 2H_para_, NHPh, *J* = 6.0 Hz); 9.33, 9.59 (s, 2H, NHPh). ^13^C NMR (DMSO-d_6_): **δ** = 113.57, 123.44, 128.20, 129.38, 130.13, 132.36, 133.25, 137.41, 152.57, 157.61 (Naphthoyl); 121.22, 124.54, 127.75, 138.45 (NHPh); 178.83 (HC=N); 189.50 (C=S).

### 2.4. Crystal Structure Determinations

Crystallographic measurements were made using an IPDS1 diffractometer (graphite monochromated Mo-K*α* radiation (*λ* = 0.71073 Å)). Data were collected using Φ scan technique with a scan width of 0.7°. The structures were solved by direct methods using the program *SIR*2004 [28] and were refined using anisotropic approximation for the nonhydrogen atoms using SHELXL-97 software [29].

### 2.5. Biological Activity 

#### 2.5.1. Cell Culture

The H460 (human lung large cell carcinoma), M-14 (human amelanotic melanoma), DU145 (human prostate carcinoma), MCF-7 (human breast adenocarcinoma), HT-29 (human colon adenocarcinoma), and K562 (human chronic myelogenous leukemia) cell lines were obtained from the research laboratory of the Faculty of Sciences and Philosophy, Universidad Peruana Cayetano Heredia. All the cells were cultured in Dulbecco's Modified Eagle Medium (DMEM) supplemented with 10% fetal calf serum and 50 **μ**g/mL gentamycin in humidified 5% CO_2_/95% air at 37°C.

### 2.6. Assessment of Cytotoxicity

The assay was performed as described previously [30]. Briefly, 3000–5000 cells were inoculated in each well of 96-well tissue culture plates and incubated at 37°C with their corresponding culture medium during 24 h. The ligands HTSC^1–5^ (10–250 *μ*M), palladium(II) complexes (0.01–10 *μ*M), or cisplatin (1–10 *μ*M) in DMSO were then added and incubated for 48 h at 37°C with a highly humidified atmosphere, 5% CO_2_ and 95% air. After the incubating period, cell monolayers were fixed with 10% trichloroacetic acid and stained for 20 minutes using the sulforhodamine B dye. Then, the excess dye was removed by washing repeatedly with 1% acetic acid. The protein-bound dye was solubilized with 10 mM Tris buffer (pH 10.5) and the absorbance values were obtained at 510 nm using a microplate reader. The IC_50_ value was defined as the concentration of a test sample resulting in a 50% reduction of absorbance as compared with untreated controls and was determined by linear regression analysis.

## 3. Results and Discussion

### 3.1. Synthesis and Characterization

**Scheme 1:**
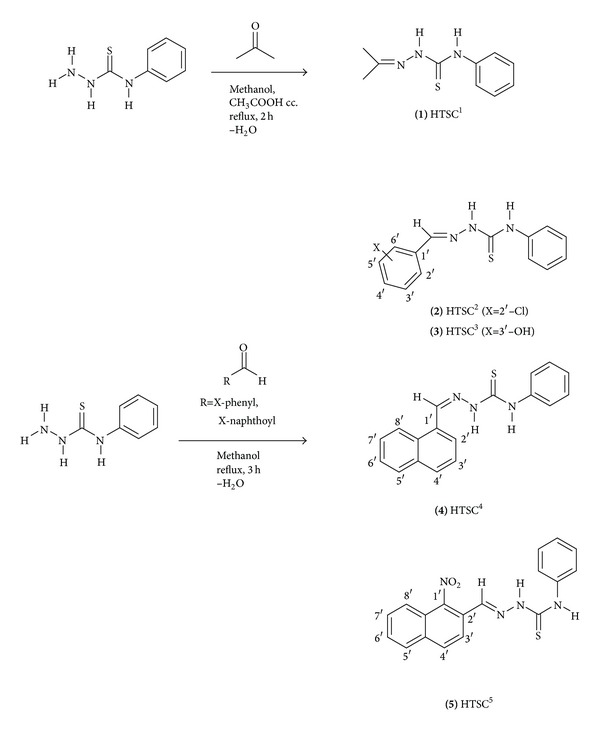
Synthesis of 4-phenyl-1-acetone thiosemicarbazone, 4-phenyl-1-benzaldehyde thiosemicarbazone, and 4-phenyl-1-naphthaldehyde thiosemicarbazone ligands.

The ligands HTSC^1–5^ were prepared according to the literature [31–33], as shown in Scheme 1. The ligands were obtained in good yields (72–87%) and characterized by elemental analysis and FT-IR, FAB(+)-mass, and NMR (^1^H, ^13^C) spectroscopy.

**Scheme 2:**
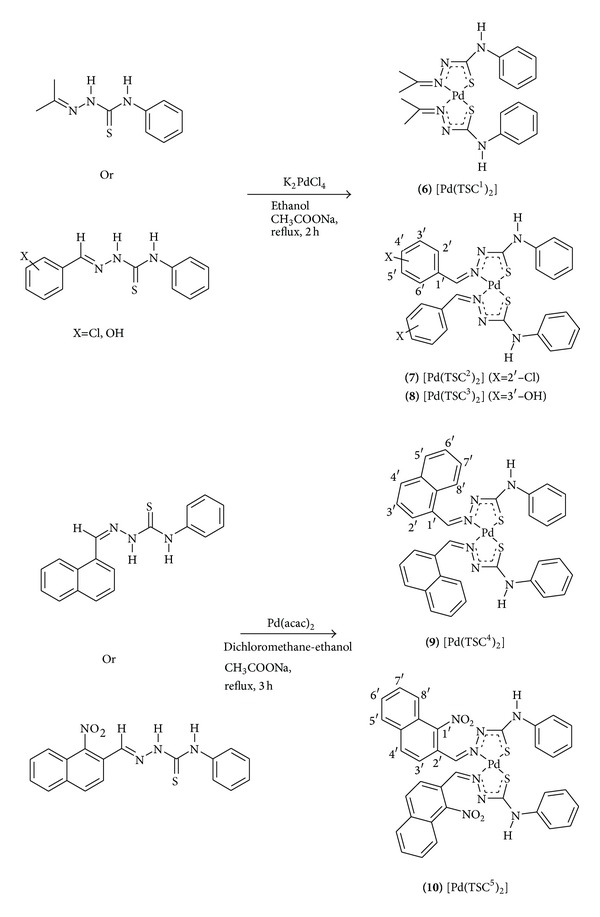
Synthesis of palladium(II) bis-chelate complexes of acetone, benzaldehyde, and naphthaldehyde thiosemicarbazone derivatives.

The palladium(II) complexes (Scheme 2) were obtained in satisfactory yield (50–68%) and characterized by elemental analysis and FT-IR, FAB(+)-mass, and NMR(^1^H, ^13^C) spectroscopy.

Analytical and spectroscopy data obtained for the thiosemicarbazone ligands and their palladium(II) complexes are in agreement with the proposed structures.

The ligand HTSC^3^ (**3**) and the complex [Pd(TSC^1^)_2_] (**6**) were recrystallized from acetone, and single crystals suitable for X-ray crystallography were obtained, while single crystals of the ligand HSTC^4^ (**4**) were obtained by slow evaporation of the solvent from the final reaction mixture.

### 3.2. Infrared Spectra

The broad bands of the –NH group observed at 3140–3182 cm^−1^ in the spectra of the free ligands disappeared in the spectra of the corresponding complexes, thus indicating the deprotonation of the =N–NH– group. The strong bands observed in the range of 1598–1626 cm^−1^ were assigned to (C=N) stretching vibrations of the free thiosemicarbazones. These bands were shifted to lower frequencies (10–22 cm^−1^) after coordination, which is in agreement with the observed behaviour of other bis-chelate complexes [26, 34–39]. These results indicate the coordination of the azomethine nitrogen to the metal ion. The **ν**(C=S) vibrations observed at 815–1088 cm^−1^ in the spectra of the free ligands shift 20–138 cm^−1^ towards lower frequencies upon complexation, indicating the involvement of the thione sulphur in the bond formation to the metal ion [40, 41].

### 3.3. NMR Spectra

The ^1^H NMR and ^13^C NMR spectra of the ligands and their metal complexes were recorded in DMSO-d_6_. In the ^1^H NMR spectra of the ligands HTSC^1–5^, the signal of the =N–NH proton appears as a singlet at **δ** 10.07–12.03, while on complexation these signals disappeared, thus indicating the deprotonation of the =N–NH group [25, 33, 42–46]. In the ^1^H NMR spectra of the ligands HTSC^2–5^, the signal of the HC=N proton appeared as a singlet at **δ** = 8.07–9.08. These signals are shifted by 0.26–0.59 ppm upfield for [Pd(TSC^2-3^)_2_] complexes (**7**, **8**). These results are consistent with the IR spectral data and suggest the coordination of palladium to the imine nitrogen [24, 25, 43–45]. For all ligands, the resonance lines found at **δ** = 9.83–10.35 were assigned to the proton of the NHPh group. The presence of the phenyl group on the terminal amine induces the shift of these signals by 1.9 ppm downfield, as compared to the resonance lines of the –NH_2_ terminal group found for other thiosemicarbazone derivatives [33, 44]. On the other hand, the aromatic proton signals of the phenyl amine group in all the ligands were observed at **δ** = 7.15–7.61, and these resonance lines show the expected calculated multiplicity. For the ligands HTSC^2^ (**2**) and HTSC^3^ (**3**) the aromatic proton signals of the phenyl fragment bound to the −CH=N group were affected by the presence of the *chloro* and *hydroxy *substituents in the C-2′ and C-3′positions, respectively, of the phenyl moiety. For the HTSC^2^ (**2**) ligand, these signals are shifted downfield for the protons in the positions C-3′ (1 ppm) and C-4′ (0.1 ppm), while for HTSC^3^ (**3**) ligand they are shifted upfield for the protons in the positions C-2′ (0.55 ppm) and C-4′ (0.21 ppm), with respect to the unsubstituted phenyl moiety [33]. For the HTSC^5^ (**5**) ligand, the presence of the *nitro* substituent group in the naphthoyl moiety affected the resonance signals of the aromatic protons. These signals are shifted downfield for the protons in the positions C-3′ (0.73 ppm) and C-6′ (0.19 ppm), while for the protons in the positions C-4′, C-5′, C-7′, and C-8′ these are shifted upfield by 0.16–0.25 ppm, relative to the HTSC^4^ (**4**) ligand with the unsubstituted naphthoyl moiety. Thus, the aromatic protons signals in all the ligands do not suffer relevant changes in their chemical shifts after complexation.

In the ^13^C NMR spectra, the carbon resonance signals of the C=N group appear at **δ** = 152.8–176.8. These results are similar to the chemical shifts found for other ligands derived from benzaldehyde thiosemicarbazone [33, 40]. The C=S signals observed at **δ** = 176.4–193.2 are characteristic for the thiocarbonyl group present in all the ligands. For [Pd(TSC^1–5^)_2_] complexes (**6**–**10**), the C=N and C=S signals are shifted downfield by 1.3–13.3 ppm and upfield by 0.5–26.1 ppm, respectively, with respect to their ligands. These results confirm the coordination of the thiocarbonyl sulphur and azomethine nitrogen atoms to the palladium(II) ion [36, 47]. For all ligands, the aromatic carbons of the NHPh group were observed at **δ** = 121.8–134.6, and these chemical shifts are in agreement with those found for other thiosemicarbazone ligands [33, 44].

### 3.4. Structural Data

Crystal data, data collection procedure, structure determination methods, and refinement results for compounds HTSC^3^, HTSC^4^, and [Pd(TSC^1^)_2_] are summarized in Table 1, whereas selected bond lengths and bond angles are presented in Tables 2 and 3.

**Figure 1:**
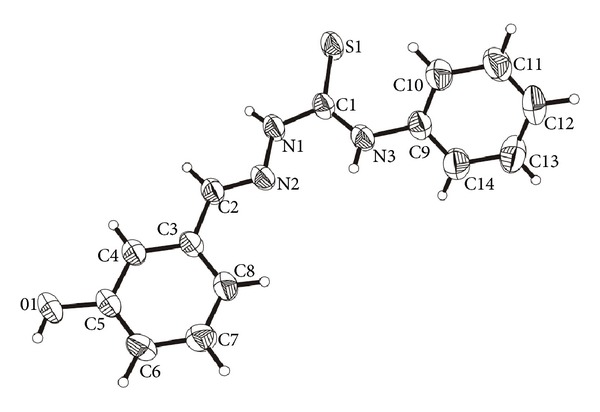
Molecular structure of HTSC^3^ (**3**). The displacement ellipsoids are drawn at the 50% probability.

**Figure 2:**
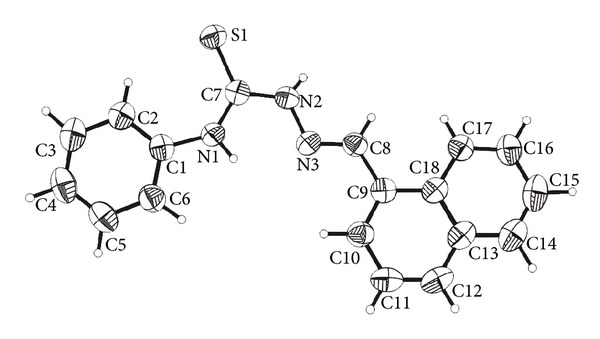
Molecular structure of HTSC^4^ (**4**). The displacement ellipsoids are drawn at the 50% probability.

**Figure 3:**
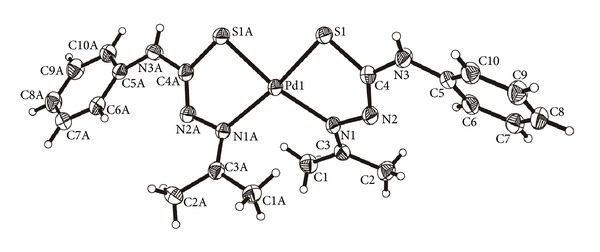
Molecular structure of [Pd(TSC^1^)_2_] (**6**). The displacement ellipsoids are drawn at the 50% probability.

The molecular structures of HTSC^3^, HTSC^4^, and [Pd(TSC^1^)_2_] are shown in Figures 1, 2, and 3, respectively. The thiocarbazone fragments in the two structures of ligands HTSC^3^ and HTSC^4^ are very similar. Structurally significant is the *cis*-arrangement between the atoms N2-N1-C1-N3 (HTSC^3^) and N3-N2-C7-N1 (HTSC^4^), the torsion angles being 5.33(2)° and 1.0(2). All bond lengths in the thiosemicarbazone fragment are identical for the two ligands (Table 2).

**Figure 4:**
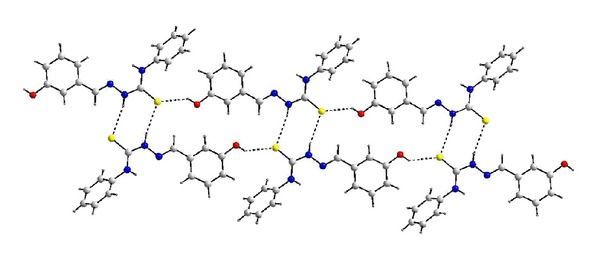
Double chain structure of HTSC^3^ in the crystal.

**Figure 5:**
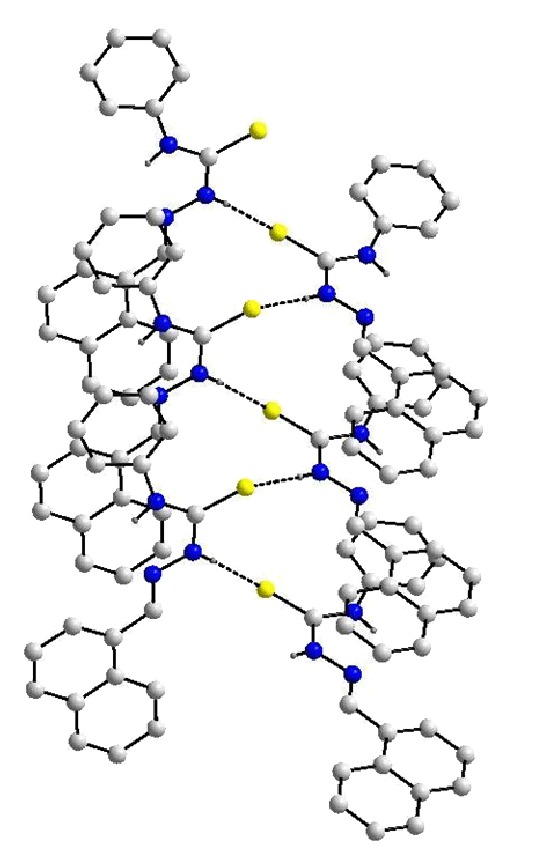
Helix structure of HTSC^4^ in the crystal.

The crystal structure of ligand HTSC^3^ is stabilized by intermolecular O–H*⋯* **S **[O*⋯*S 3.169 Å, H*⋯*S 2.548 Å, and O–H*⋯*S 136.46°] hydrogen bonds which lead to a double chain along the a-axis, as shown in Figure 4. On the other hand, the crystal structure of ligand HTSC^4^ is also stabilized by a N–H*⋯* **S **hydrogen bond with the bond parameters as follows: N*⋯*S 3.523 Å, H*⋯*S 3.523 Å, and N–H–S 158.82°. We found along of a 2_1_-screw axis a typical helix structure, as shown in Figure 5.

The complex [Pd(TSC^1^)_2_] (**6**) (Figure 3) crystallizes in the monoclinic space group C2/c with four molecules in the unit cell and with a C_2_ molecular symmetry. The sulfur and nitrogen donor atoms are in a *cis* arrangement. The deprotonated ligand coordinates bidentately to Pd^II^ ion through S and N. It leads to lengthening of the C4–S1 bond (1.773 Å) and shortening of the N2–C4 bond (1.29 Å) and these results are in agreement with those found for other palladium(II) bis-chelate complexes of the type [PdL_2_] with thiosemicarbazone ligands [27, 42].

The chelate ring with the atoms Pd1, N1, N2, C4, and S1 has an envelope configuration. For the plane formed by the atoms N1, N2, S1, and C4, the average deviation is 0.003 Å, while the deviation of the Pd atom from this plane is 0.664 Å; this distortion indicates a pseudo square planar coordination geometry.

### 3.5. Antitumor Evaluation

The cytotoxic potential of the ligands derived from thiosemicarbazones and their respective palladium(II) complexes were investigated in the following six human tumor cell lines: H460, DU145, MCF-7, M14, HT-29, and K562. For comparison purposes, the cytotoxicity of cisplatin was evaluated under the same experimental conditions.

**Figure 6:**
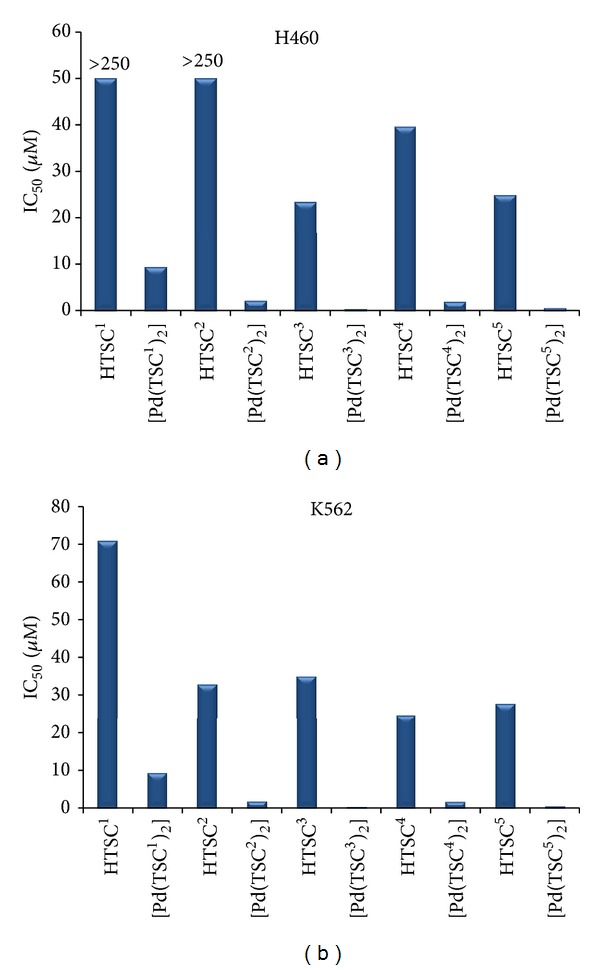
The antiproliferative activity *in vitro* expressed as IC_50_ (*μ*M) values of the ligands HTSC^1–5^ and palladium(II) complexes [Pd(TSC^1–5^)_2_] against (a) H460 and (b) K562 human tumor cell lines after 48 h incubation time.

The results of the cytotoxic activity of the ligands, palladium(II) complexes, and cisplatin are expressed as IC_50_ values (micromolar concentration inhibiting 50% cell growth), and these compounds were evaluated *in vitro* against the different human tumor cell lines, as shown in Table 4. In general, the palladium(II) complexes (IC_50_ = 0.01–9.87 *μ*M) exhibited higher antiproliferative activity than their free ligands (IC_50_ = 23.48–70.86 and >250 *μ*M).  Figure 6 shows the antiproliferative activity of the ligands HTSC^1–5^ and their palladium(II) complexes [Pd(TSC^1–5^)_2_] against H460 and K562 human tumor cell lines after 48 h incubation time. These results indicate that the cytotoxicity is enhanced when the ligands are coordinated to the Pd(II) ion. Probably, the palladium(II) bis-chelate complexes of square planar geometry act as intercalating agents between the pyrimidine and guanine bases of the DNA tumor cells, inducing conformational changes on the DNA double helix specific that finally produce tumor cell death [33, 44, 48].

**Figure 7:**
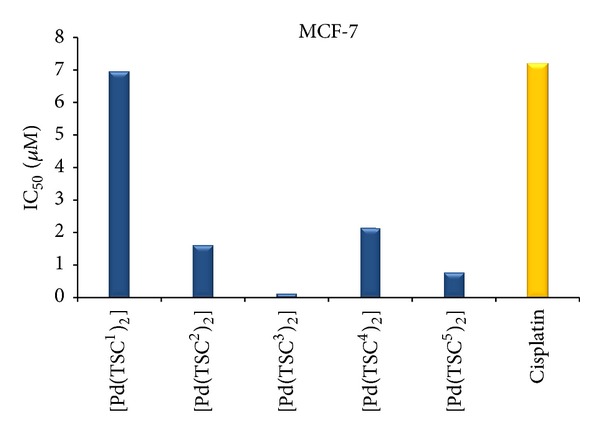
The antiproliferative activity *in vitro* expressed as IC_50_ (*μ*M) values of the ligands HTSC^1–5^, palladium(II) complexes [Pd(TSC^1–5^)_2_], and cisplatin against MCF-7 human tumor cell line after 48 h incubation time.

All palladium(II) complexes except [Pd(TSC^1^)_2_] (**6**) were more cytotoxic than cisplatin (IC_50_ = 2.85−7.60 *μ*M) against all the investigated human tumor cell lines.  Figure 7 shows a comparison of the magnitude of the IC_50_ values of the palladium(II) complexes and cisplatin against human breast adenocarcinoma MCF-7 cell line. On the other hand, between all the tested palladium(II) complexes, [Pd(TSC^3^)_2_] (**8**) and [Pd(TSC^5^)_2_] (**10**) complexes showed greater cytotoxic activity against all human tumor cell lines, with IC_50_ values of 0.01–0.23 and 0.65–1.06 *μ*M, respectively. Therefore, the presence of the 3-*hydroxy* and 1-*nitro* substituents groups in the benzene and naphthalene aromatic rings plays an important role in the enhancement the antiproliferative activity [1, 16, 27, 42]. The effect of these substituents may be related to their hydrogen-bonding ability compared with the chloro substituent in complex (**7**). Following this reasoning, the [Pd(TSC^3^)_2_] (**8**) complex, with the 4-phenyl-1-(3′-hydroxy-benzaldehyde) thiosemicarbazone ligand, was also more active than the palladium(II) bis-chelate complex of 3′-cyano-benzaldehyde thiosemicarbazone (IC_50_ = 0.45–3.53 *μ*M) against all human tumor cell lines tested [27]. In addition, complex (**8**) was found to be about thirteen times more cytotoxic than the gold(I) complex from 4-methyl-1-(2′-acetylpyridine) thiosemicarbazone ligand (IC_50_ = 1.65 *μ*M) against the (MCF-7) human breast adenocarcinoma tumor cell line [49]. With respect to the cytotoxic activity shown by the ruthenium(II) complex of the [Ru(Phen)_2_(L)]Cl_2_ type, with L being a 3-methoxy, or 4-hydroxy-benzaldehyde thiosemicarbazone ligand (IC_50_ = 3.60 *μ*M) assayed on the (CEM) human leukemia cell line [24], complex (**8**) presented higher cytotoxicity at low micromolar concentrations (IC_50_ = 0.02 *μ*M) tested *in vitro* against the (K562) chronic myelogenous leukemia cell line. Since the ruthenium complex is octahedral and complex (**8**) presents a pseudo-planar geometry, the larger cytotoxicity of (**8**) is in agreement with the proposed intercalation mechanism as the intercalation is favored for a planar moiety.

Complex [Pd(TSC^5^)_2_] (**10**) (IC_50_ = 0.78 *μ*M) tested against the MCF-7 tumor cell line resulted to be more cytotoxic than the palladium(II) monochelate complexes with 2-acetylpyridine thiosemicarbazone derivatives (IC_50_ = 4.9–5.5 *μ*M) when being tested on the MDA-MB231 human breast cancer cell line [32]. Furthermore, complex (**10**) tested *in vitro* against the HT29 colon adenocarcinoma tumor cell line exhibited higher cytotoxicity (IC_50_ = 1.04 *μ*M) than that of the [Pd(mpETSC) Cl] (HmpETSC = 4-ethyl-1-(6′-methylpyridine-2′-carbaldehyde) thiosemicarbazone) monochelate complex (IC_50_ = 20.65 **μ**M) assayed on the HCT 116 human colon tumor cell line [26].

In summary, we have synthesized palladium(II) bis-chelate complexes with ligands derived from acetone, benzaldehyde, and naphthaldehyde thiosemicarbazone. The molecular structure of [Pd(TSC^1^)_2_] (**6**) shows a square-planar geometry with deprotonated ligands coordinated to Pd(II) through the azomethine nitrogen and thione sulfur atoms in a *cis *arrangement.

Of all the studied complexes, the hydroxy-substituted [Pd(TSC^3^)_2_] (**8**) complex resulted to be more cytotoxic in all tumor cell lines at low micromolar concentrations, compared to the other complexes and the free ligands.

### 3.6. Extra Material

Crystallographic data for the structural analysis have been deposited with the Cambridge Crystallographic Data Centre, numbers CCDC 894930 for HTSC^3^, 894931 for [Pd(TSC^1^)_2_], and 894932 for HTSC^4^. Copies of this information can be obtained free of charge from the Cambridge Crystallographic Data Center (CCDC, 12 Union Road, Cambridge CB2 1EZ, UK; fax: (+44) 1223-336-033; email: deposit@ccdc.cam.ac.uk).

## Supplementary Material

Supplementary Information (figures containing IR, ^1^H, COSY and ^13^C NMR spectra).

## Figures and Tables

**Table 1:** Crystal data and structure refinement for HTSC^3^, HTSC^4^, and [Pd(TSC^1^)_2_].

Compound	HTSC^3^	HTSC^4^	[Pd(TSC^1^)_2_]
Empirical formula	C_14_H_13_N_3_OS	C_18_H_15_N_3_S	C_20_H_24_N_6_S_2_Pd
Formula weight	271.33	305.39	518.97
Temperature (K)	213	213	213
Crystal system	Triclinic	Orthorhombic	Monoclinic
Space group	P-1	P2_1_2_1_2_1_	C2/c
*a* (Å)	6.3202(7)	5.3471(3)	23.456(2)
*b* (Å)	10.357(1)	15.7563(9)	7.7080(4)
*c* (Å)	11.506(1)	16.456(2)	12.3813(10)
*α* (°)	65.95(1)	90	90
*β* (°)	80.14(1)	90	97.96(1)
*γ* (°)	85.32(1)	90	90
Volume (Å^3^)	677.56(13)	1554.91(18)	2216.9(3)
*Z*	2	4	4
Density (g/cm^3^)	1.33	1.305	1.555
Absorption coeff. (mm^−1^)	0.234	0.208	1.044
Crystal size (mm)	0.7 × 0.3 × 0.2	0.7 × 0.05 × 0.05	0.4 × 0.4 × 0.4
*θ* range for data collect. (°)	3–28	2–26	3–28
Index ranges	−7 ≤ *h* ≤ 8	− 6 ≤ *h* ≤ 6	− 30 ≤ *h* ≤ 30
	−13 ≤ *k* ≤ 13	− 18 ≤ *k* ≤ 19	−9 ≤ *k* ≤ 10
	−15 ≤ *l* ≤ 15	− 22 ≤ *l* ≤ 22	−16 ≤ *l* ≤ 16
Reflections collected	7186	10232	10366
Independent reflections	2997 (*R*_int⁡_ = 0.024)	3005 (*R*_int⁡_ = 0.050)	2662 (*R*_int⁡_ = 0.042)
Max./min. transmission	0.8534/0.9547	0.8683/0.9897	0.7171/0.9674
Data/parameters	2997/224	3005/259	2662/180
Goodness-of-fit on *F* ^2^	0.803	0.804	0.841
Final *R* indices [*I* > 2*σ*(*I*)]	*R*_1_ = 0.0289	*R*_1_ = 0.0322	*R*_1_ = 0.0235
	*ωR*_2_ = 0.0685	*ωR*_2_ = 0.0624	*ωR*_2_ = 0.0442
*R* indices (all data)	*R*_1_ = 0.0478	*R*_1_ = 0.0486	*R*_1_ = 0.0317
	*ωR*_2_ = 0.0717	*ωR*_2_ = 0.0653	*ωR*_2_ = 0.0454
Lgst diff. peak/hole (*e*Å^−3^)	0.19/−0.16	−0.1/0.03	−0.31/0.07

**Table 2:** Bond length (Å) and torsion angles (°) for HTSC^3^ and HTSC^4^.

HTSC^3^	HTSC^4^
C2-N2 1.280(2)	C8-N3 1.278(2)
N2-N1 1.382(2)	N3-N2 1.380(2)
N1-C1 1.352(2)	N2-C7 1.351(2)
C1-N3 1.347(2)	C7-N1 1.351(2)
C1-S1 1.689(1)	C7-S1 1.688(2)
N3-C9 1.430(2)	N1-C1 1.414(2)
C2-N2-N1-C1 173.8(1)	C8-N3-N2-C7 180.0(2)
N2-N1-C1-N3 5.33(2)	N3-N2-C7-N1 1.0(2)
N1-C1-N3-C9 −177.1(1)	N2-C7-N1-C1 −176.8(2)

**Table 3:** Selected bond lengths (Å) and angles in (°) for [Pd(TSC^1^)_2_] (**6**).

Distances	Angles
Pd1-S1 2.270(1)	N1-Pd1-S1 82.21(4)
Pd1-N1 2.099(1)	Pd1-S1-C4 95.20(6)
N1-N2 1.421(2)	S1-C4 -N2 125.7(1)
N2-C4 1.290(2)	C4-N2-N1 112.6(2)
C4-S1 1.773(2)	N2-N1-Pd1 117.3(1)

**Table 4:** IC_50_ (*µ*m) values^a^ of the ligands HTSC^1–5^, palladium(II) complexes [Pd(TSC^1–5^)_2_], and cisplatin against the different human tumor cell lines^b^.

Human tumor cell lines	H460	DU145	MCF-7	M14	HT-29	K562
HTSC^1^	>250	>250	>250	>250	>250	70.86
HTSC^2^	>250	31.55	38.05	>250	>250	32.89
HTSC^3^	23.48	26.64	34.00	28.67	25.73	35.05
HTSC^4^	39.65	26.45	29.94	>250	>250	24.66
HTSC^5^	24.95	31.60	25.46	27.31	26.72	27.76
[Pd(TSC^1^)_2_]	9.40	8.27	6.95	9.87	8.20	9.43
[Pd(TSC^2^)_2_]	2.26	2.05	1.61	2.14	1.87	1.95
[Pd(TSC^3^)_2_]	0.23	0.01	0.13	0.05	0.05	0.02
[Pd(TSC^4^)_2_]	2.05	2.39	2.14	2.27	2.37	1.84
[Pd(TSC^5^)_2_]	0.68	0.84	0.78	1.06	1.04	0.65
cisplatin	2.85	6.50	7.20	2.95	7.60	3.20

^a^IC_50_ corresponds to the concentration required to inhibit 50% of the cell growth when the cells are exposed to the compounds during 48 h. Each value is the average of two independent experiments.

^b^Lung large cell carcinoma (H460), prostate carcinoma (DU145), breast adenocarcinoma (MCF-7), amelanotic melanoma (M-14), colon adenocarcinoma (HT-29), and chronic myelogenous leukemia (K562).
