# Supplementary material for: Synthesis and Characterization of New Palladium(II) Thiosemicarbazone Complexes and Their Cytotoxic Activity against Various Human Tumor Cell Lines
Source: Bioinorg Chem Appl. 2013 Dec 11;2013:524701. doi: 10.1155/2013/524701 (PMC3874341; doi:10.1155/2013/524701)

checkCIF/PLATON report

You have not supplied any structure factors. As a result the full set of tests cannot be run.

No syntax errors found.   CIF dictionary   Interpreting this report

Datablock: ip1_996a

```
Bond precision:  C-C = 0.0023 A          Wavelength=0.71073

Cell:           a=6.3202(7)        b=10.3566(11)     c=11.5058(13)
                alpha=65.954(12)   beta=80.144(13)   gamma=85.320(13)
Temperature:    213 K

                  Calculated                 Reported
Volume            677.56(15)                 677.56(13)
Space group       P -1                       P-1
Hall group        -P 1                       ?
Moiety formula    C14 H13 N3 O S             ?
Sum formula       C14 H13 N3 O S             C14 H13 N3 O S
Mr                271.34                     271.33
Dx,g cm-3         1.330                      1.330
Z                 2                          2
Mu (mm-1)         0.234                      0.234
F000              284.0                      284.0
F000'             284.34
h,k,lmax          8,13,15                    8,13,15
Nref              3273                       2997
Tmin,Tmax         0.919,0.954                0.853,0.955
Tmin'             0.849

Correction method= NONE

Data completeness= 0.916          Theta(max)= 27.970

R(reflections)= 0.0289( 1904)     wR2(reflections)= 0.0717( 2997)

S = 0.803                    Npar= 224
```

The following ALERTS were generated. Each ALERT has the format
test-name_ALERT_alert-type_alert-level.
Click on the hyperlinks for more details of the test.

Alert level A

PLAT029_ALERT_3_A _diffrn_measured_fraction_theta_full Low 0.916

Alert level C

```
ABSTY03_ALERT_1_C  The _exptl_absorpt_correction_type has been given as none.
            However values have been given for Tmin and Tmax. Remove
            these if an absorption correction has not been applied.
  From the CIF: _exptl_absorpt_correction_T_min   0.853
  From the CIF: _exptl_absorpt_correction_T_max   0.955
```

Alert level G

```
PLAT005_ALERT_5_G No _iucr_refine_instructions_details  in the CIF          ?
PLAT063_ALERT_4_G Crystal Size Likely too Large for Beam Size ....       0.70 mm
PLAT152_ALERT_1_G The Supplied and Calc. Volume s.1. Differ by ...          2 Units
```

```
   1 ALERT level A = Most likely a serious problem - resolve or explain
   0 ALERT level B = A potentially serious problem, consider carefully
   1 ALERT level C = Check. Ensure it is not caused by an omission or oversight
   3 ALERT level G = General information/check it is not something unexpected

   2 ALERT type 1 CIF construction/syntax error, inconsistent or missing data
   0 ALERT type 2 Indicator that the structure model may be wrong or deficient
   1 ALERT type 3 Indicator that the structure quality may be low
   1 ALERT type 4 Improvement, methodology, query or suggestion
   1 ALERT type 5 Informative message, check
```

It is advisable to attempt to resolve as many as possible of the alerts in all categories. Often the minor alerts point to easily fixed oversights, errors and omissions in your CIF or refinement strategy, so attention to these fine details can be worthwhile. In order to resolve some of the more serious problems it may be necessary to carry out additional measurements or structure refinements. However, the purpose of your study may justify the reported deviations and the more serious of these should normally be commented upon in the discussion or experimental section of a paper or in the "special_details" fields of the CIF. checkCIF was carefully designed to identify outliers and unusual parameters, but every test has its limitations and alerts that are not important in a particular case may appear. Conversely, the absence of alerts does not guarantee there are no aspects of the results needing attention. It is up to the individual to critically assess their own results and, if necessary, seek expert advice.

Publication of your CIF in IUCr journals

A basic structural check has been run on your CIF. These basic checks will be run on all CIFs submitted for publication in IUCr journals (*Acta Crystallographica*, *Journal of Applied Crystallography*, *Journal of Synchrotron Radiation*); however, if you intend to submit to *Acta Crystallographica Section C* or *E*, you should make sure that full publication checks are run on the final version of your CIF prior to submission.

Publication of your CIF in other journals

Please refer to the *Notes for Authors* of the relevant journal for any special instructions relating to CIF submission.

PLATON version of 05/11/2012; check.def file version of 05/11/2012

Datablock ip1_996a - ellipsoid plot

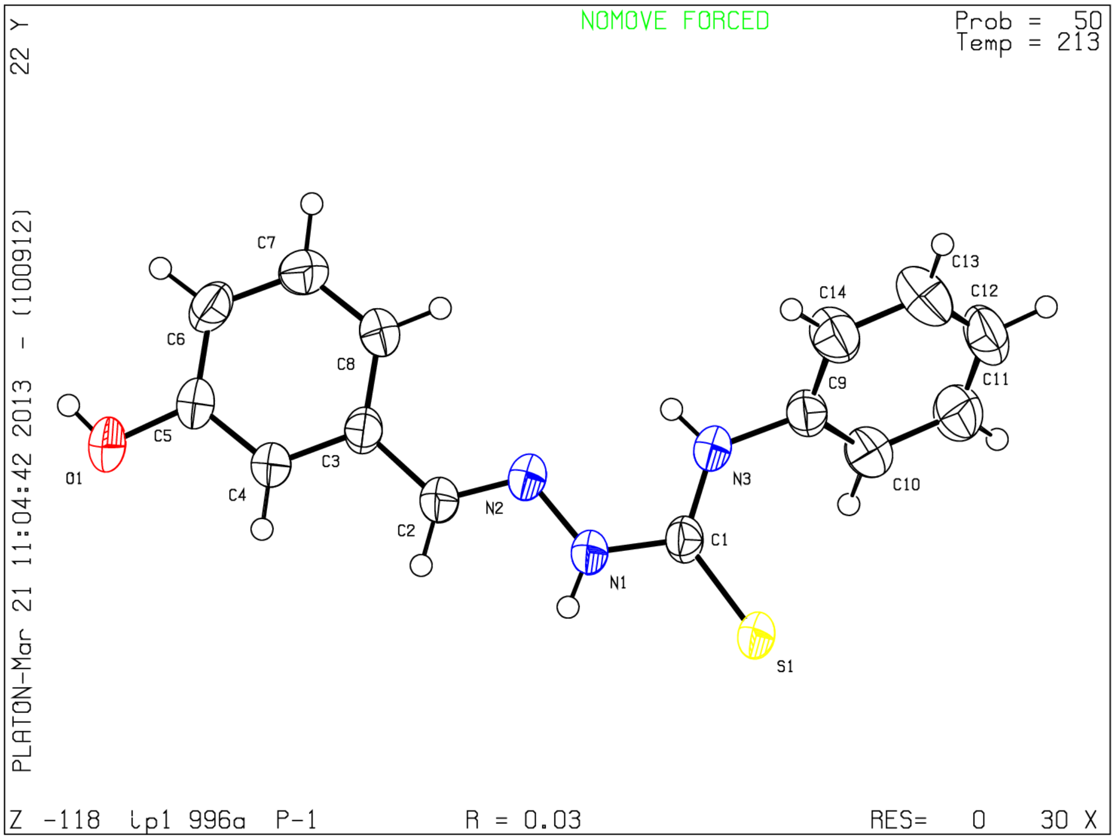

Supplement: Supplementary file 1 — Supplementary Information (figures containing IR, 1H, COSY and 13C NMR spectra). [file 524701.f1.pdf]
