# Supplementary material for: Synthesis and Characterization of New Palladium(II) Thiosemicarbazone Complexes and Their Cytotoxic Activity against Various Human Tumor Cell Lines
Source: Bioinorg Chem Appl. 2013 Dec 11;2013:524701. doi: 10.1155/2013/524701 (PMC3874341; doi:10.1155/2013/524701)

checkCIF/PLATON report

You have not supplied any structure factors. As a result the full set of tests cannot be run.

No syntax errors found. CIF dictionary Interpreting this report

Datablock: ip1019a

```
Bond precision:  C-C = 0.0030 A          Wavelength=0.71073

Cell:           a=23.4555(19)    b=7.7080(4)      c=12.3813(10)
                alpha=90         beta=97.962(9)   gamma=90
Temperature:    213 K

                Calculated                 Reported
Volume          2216.9(3)                  2216.9(3)
Space group     C 2/c                      C2/c
Hall group      -C 2yc                     ?
Moiety formula  C20 H24 N6 Pd S2           ?
Sum formula     C20 H24 N6 Pd S2           C20 H24 N6 Pd S2
Mr              518.99                     518.97
Dx,g cm-3       1.555                      1.555
Z               4                          4
Mu (mm-1)       1.044                      1.044
F000            1056.0                     1056.0
F000'           1053.23
h,k,lmax        30,10,16                   30,10,16
Nref            2679                       2662
Tmin,Tmax       0.694,0.959                0.717,0.967
Tmin'           0.652

Correction method= NUMERICAL

Data completeness= 0.994          Theta(max)= 27.990

R(reflections)= 0.0235( 2143)     wR2(reflections)= 0.0454( 2662)

S = 0.841                   Npar= 180
```

The following ALERTS were generated. Each ALERT has the format
test-name_ALERT_alert-type_alert-level.
Click on the hyperlinks for more details of the test.

Alert level C

ABSTY02_ALERT_1_C An _exptl_absorpt_correction_type has been given without a literature citation. This should be contained in the _exptl_absorpt_process_details field.
Absorption correction given as numerical

```
PLAT350_ALERT_3_C Short   C-H Bond (0.96A)   C1     -   H1B    ...       0.83 Ang.
PLAT391_ALERT_3_C Deviating Methyl C2      H-C-H Bond Angle ......        101 Deg.
PLAT420_ALERT_2_C D-H Without Acceptor       N3     -   H3N    ...          ?
```

Alert level G

```
PLAT005_ALERT_5_G No _iucr_refine_instructions_details  in the CIF          ?
PLAT164_ALERT_4_G Nr. of Refined C-H H-Atoms in Heavy-Atom Struct.         11
```

```
   0 ALERT level A = Most likely a serious problem - resolve or explain
   0 ALERT level B = A potentially serious problem, consider carefully
   4 ALERT level C = Check. Ensure it is not caused by an omission or oversight
   2 ALERT level G = General information/check it is not something unexpected

   1 ALERT type 1 CIF construction/syntax error, inconsistent or missing data
   1 ALERT type 2 Indicator that the structure model may be wrong or deficient
   2 ALERT type 3 Indicator that the structure quality may be low
   1 ALERT type 4 Improvement, methodology, query or suggestion
   1 ALERT type 5 Informative message, check
```

It is advisable to attempt to resolve as many as possible of the alerts in all categories. Often the minor alerts point to easily fixed oversights, errors and omissions in your CIF or refinement strategy, so attention to these fine details can be worthwhile. In order to resolve some of the more serious problems it may be necessary to carry out additional measurements or structure refinements. However, the purpose of your study may justify the reported deviations and the more serious of these should normally be commented upon in the discussion or experimental section of a paper or in the "special_details" fields of the CIF. checkCIF was carefully designed to identify outliers and unusual parameters, but every test has its limitations and alerts that are not important in a particular case may appear. Conversely, the absence of alerts does not guarantee there are no aspects of the results needing attention. It is up to the individual to critically assess their own results and, if necessary, seek expert advice.

Publication of your CIF in IUCr journals

A basic structural check has been run on your CIF. These basic checks will be run on all CIFs submitted for publication in IUCr journals (*Acta Crystallographica*, *Journal of Applied Crystallography*, *Journal of Synchrotron Radiation*); however, if you intend to submit to *Acta Crystallographica Section C* or *E*, you should make sure that full publication checks are run on the final version of your CIF prior to submission.

Publication of your CIF in other journals

Please refer to the *Notes for Authors* of the relevant journal for any special instructions relating to CIF submission.

PLATON version of 05/11/2012; check.def file version of 05/11/2012

Datablock ip1019a - ellipsoid plot

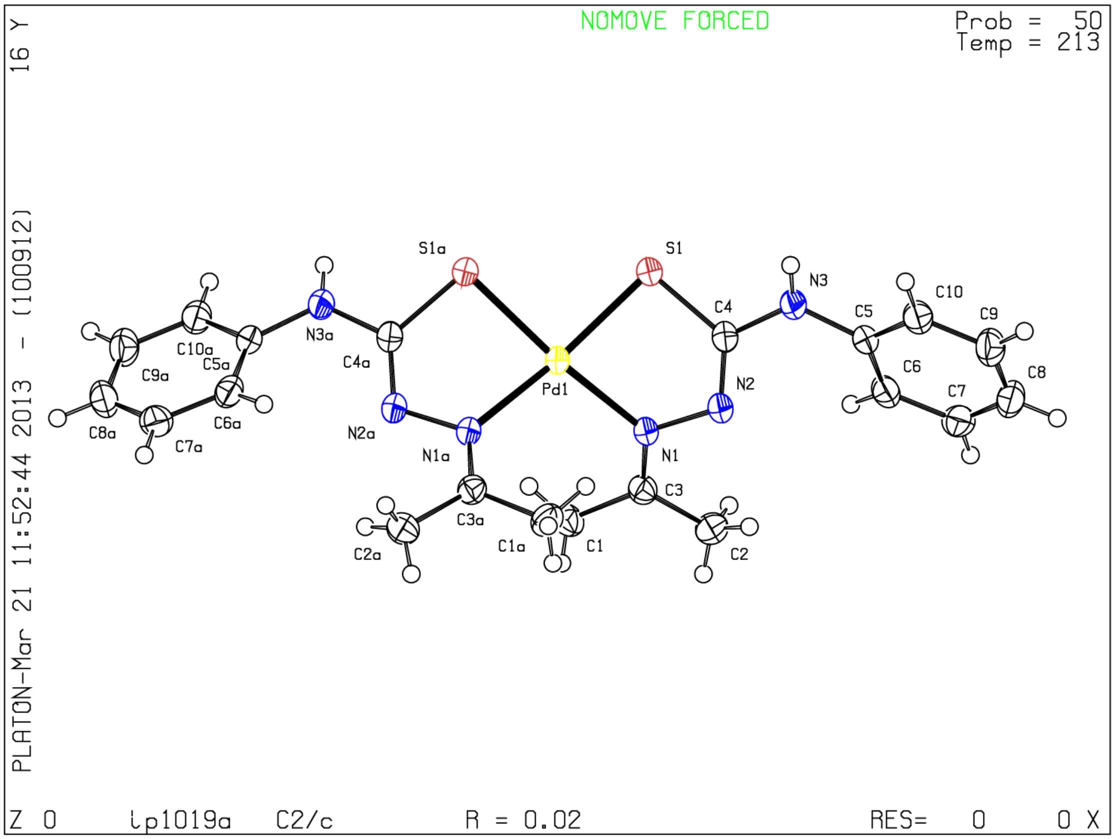

Supplement: Supplementary file 2 [file 524701.f2.pdf]
