# Supplementary material for: Synthesis and Characterization of New Palladium(II) Thiosemicarbazone Complexes and Their Cytotoxic Activity against Various Human Tumor Cell Lines
Source: Bioinorg Chem Appl. 2013 Dec 11;2013:524701. doi: 10.1155/2013/524701 (PMC3874341; doi:10.1155/2013/524701)

checkCIF/PLATON report

You have not supplied any structure factors. As a result the full set of tests cannot be run.

No syntax errors found. CIF dictionary Interpreting this report

Datablock: ip1026

```
Bond precision:  C-C = 0.0032 A          Wavelength=0.71073

Cell:            a=5.3471(3)     b=15.7563(9)     c=18.4558(15)
                 alpha=90        beta=90          gamma=90
Temperature:     213 K

                Calculated                  Reported
Volume          1554.91(18)                 1554.91(18)
Space group     P 21 21 21                  P2(1)2(1)2(
Hall group      P 2ac 2ab                   ?
Moiety formula  C18 H15 N3 S                ?
Sum formula     C18 H15 N3 S                C18 H15 N3 S
Mr              305.40                      305.39
Dx,g cm-3       1.305                       1.305
Z               4                           4
Mu (mm-1)       0.208                       0.208
F000            640.0                       640.0
F000'           640.68
h,k,lmax        6,19,22                     6,19,22
Nref            1782[ 3026]                 3005
Tmin,Tmax       0.988,0.990                 0.868,0.990
Tmin'           0.865

Correction method= NONE

Data completeness= 1.69/0.99      Theta(max)= 25.850

R(reflections)= 0.0322( 2151)     wR2(reflections)= 0.0653( 3005)

S = 0.804                   Npar= 259
```

The following ALERTS were generated. Each ALERT has the format
test-name_ALERT_alert-type_alert-level.
Click on the hyperlinks for more details of the test.

Alert level B

SYMMS01_ALERT_1_B The cell setting should be one of the following
- triclinic
- monoclinic
- orthorhombic

```
            *  tetragonal
            *  rhombohedral
            *  trigonal
            *  hexagonal
            *  cubic
            Cell setting given = orthorombic
```

Alert level C

```
ABSTY03_ALERT_1_C  The _exptl_absorpt_correction_type has been given as none.
            However values have been given for Tmin and Tmax. Remove
            these if an absorption correction has not been applied.
  From the CIF: _exptl_absorpt_correction_T_min   0.868
  From the CIF: _exptl_absorpt_correction_T_max   0.990
PLAT230_ALERT_2_C Hirshfeld Test Diff for    S1     --  C7      ..        6.4 su
PLAT230_ALERT_2_C Hirshfeld Test Diff for    C15    --  C16     ..        6.5 su
PLAT245_ALERT_2_C U(iso) H2N     Smaller than U(eq) N2      by ...      0.016 AngSq
PLAT245_ALERT_2_C U(iso) H8      Smaller than U(eq) C8      by ...      0.023 AngSq
PLAT245_ALERT_2_C U(iso) H17     Smaller than U(eq) C17     by ...      0.014 AngSq
PLAT352_ALERT_3_C Short   N-H Bond (0.87A)   N2     -   H2N    ...       0.75 Ang.
PLAT790_ALERT_4_C Centre of Gravity not Within Unit Cell: Resd.  #          1
              C18 H15 N3 S
```

Alert level G

```
PLAT005_ALERT_5_G No _iucr_refine_instructions_details  in the CIF          ?
PLAT063_ALERT_4_G Crystal Size Likely too Large for Beam Size ....       0.70 mm
PLAT104_ALERT_1_G The Reported Crystal System is Inconsistent with   P212121
```

   0 **ALERT level A** = Most likely a serious problem - resolve or explain
   1 **ALERT level B** = A potentially serious problem, consider carefully
   8 **ALERT level C** = Check. Ensure it is not caused by an omission or oversight
   3 **ALERT level G** = General information/check it is not something unexpected

   3 ALERT type 1 CIF construction/syntax error, inconsistent or missing data
   5 ALERT type 2 Indicator that the structure model may be wrong or deficient
   1 ALERT type 3 Indicator that the structure quality may be low
   2 ALERT type 4 Improvement, methodology, query or suggestion
   1 ALERT type 5 Informative message, check

It is advisable to attempt to resolve as many as possible of the alerts in all categories. Often the minor alerts point to easily fixed oversights, errors and omissions in your CIF or refinement strategy, so attention to these fine details can be worthwhile. In order to resolve some of the more serious problems it may be necessary to carry out additional measurements or structure refinements. However, the purpose of your study may justify the reported deviations and the more serious of these should normally be commented upon in the discussion or experimental section of a paper or in the "special_details" fields of the CIF. checkCIF was carefully designed to identify outliers and unusual parameters, but every test has its limitations and alerts that are not important in a particular case may appear. Conversely, the absence of alerts does not guarantee there are no aspects of the results needing attention. It is up to the individual to critically assess their own results and, if necessary, seek expert advice.

Publication of your CIF in IUCr journals

A basic structural check has been run on your CIF. These basic checks will be run on all CIFs submitted for publication in IUCr journals (*Acta Crystallographica*, *Journal of Applied Crystallography*, *Journal of Synchrotron Radiation*); however, if you intend to submit to *Acta Crystallographica Section C* or *E*, you should make sure that full publication checks are run on the final version of your CIF prior to submission.

Publication of your CIF in other journals

Please refer to the *Notes for Authors* of the relevant journal for any special instructions relating to CIF submission.

PLATON version of 05/11/2012; check.def file version of 05/11/2012

Datablock ip1026 - ellipsoid plot

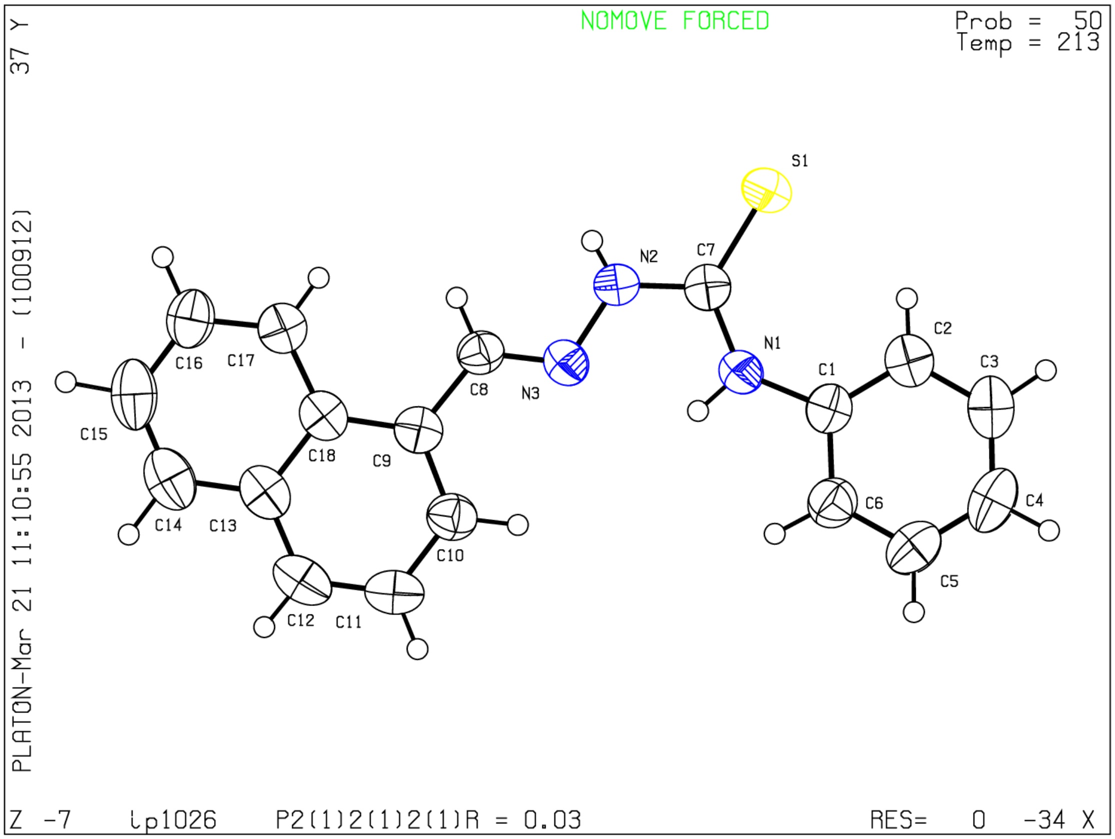

Supplement: Supplementary file 3 [file 524701.f3.pdf]
